# Rosai-Dorfman disease: A pathological experience from 2 tertiary care hospitals in Western Saudi Arabia

**DOI:** 10.15537/smj.2022.43.4.20220028

**Published:** 2022-04

**Authors:** Jaudah A. Al-Maghrabi

**Affiliations:** *From the Department of Pathology, King Abdulaziz University, and from the Department of Pathology and Laboratory Medicine, King Faisal Specialist Hospital and Research Center, Jeddah, Kingdom of Saudi Arabia.*

**Keywords:** Rosai-Dorfman disease, sinus histiocytosis, massive lymphadenopathy, Saudi Arabia

## Abstract

**Objectives:**

To document the clinicopathological features of Rosai-Dorfman disease (RDD) at 2 tertiary hospitals in the western region of Saudi Arabia.

**Methods:**

We retrieved all cases diagnosed as RDD at King Abdulaziz University Hospital and King Faisal Specialist Hospital and Research Center, Jeddah, Saudi Arabia, diagnosed between January 2001 until June 2021.

**Results:**

A total of 13 new RDD cases were reported, including 7 nodal and 6 extranodal type. The extranodal sites included larynx, optic chiasm, dura and brain, lumbar vertebrae, and left arm soft tissue.

There were 6 males and 7 females. Ages averaged 34 years and ranged from 4-56 years. A total of 2 cases were associated with Hodgkin’s lymphoma, and 2 cases have been initially misdiagnosed as other entities. All patients were treated with surgical excision, and steroid was added in 3 cases. Over 2-60 months of follow-up, recurrence occurred in 2 cases.

**Conclusion:**

Awareness of this entity is important for pathologists to avoid misdiagnosis. While the optimal treatment remains controversial, surgical resection is generally curative. The prognosis is good with rare recurrence. Multicenteer prospective studies are probably the best to evaluate treatment options and improve outcomes.

**R**osai-Dorfman disease (RDD) is a rare benign idiopathic histiocytic lesion with heterogeneous clinical features. Generally, it is a self-limiting condition involving nodal and extranodal sites. It may be familial or associated with neoplasia or immune disease. Rosai-Dorfman disease is also known as sinus histiocytosis with massive lymphadenopathy. It usually affects young patients and produces prominent bilateral cervical lymphadenopathy. Extranodal RDD accounts for approximately 25-40% of all cases.^1-3^ Rosai-Dorfman disease in particularly isolated extranodal forms can represent a diagnostic challenge for the unaware anatomic pathologist.

The pattern of the disease and the sites of involvement differ for ethnic groups. Elsheikh et al^4^ found that the most common sites for RDD varies among different ethnic groups. It was found that for African Americans, the most common sites were lymph nodes (LNs) and superficial soft tissues. For whites, the central nervous system (CNS) and maxillofacial sites were the most common, while LNs and subcutaneous tissue were the most common sites among Asian in their study.

Brenn et al^5^ and Wang et al^6^ analyzed the ethnic background for cutaneous RDD. They demonstrated a significant white and Asian predominance compared to black. These findings were in contrast with the classic nodal RDD, which usually affects children and has a propensity for whites and blacks compared to other races.^7^ Wang et al^6^ demonstrated a clear distinction between cutaneous RDD and systemic RDD. Cutaneous RDD affects older age groups, has a different male:female ratio, and affects different ethnic groups than the systemic RDD. Therefore, RDD seems to be a heterogeneous disease with racial and ethnic influence.

Little is known regarding the pattern of RDD and common sites of involvement in Saudi Arabia, and information is limited to case reports. This study aims to review all the cases of RDD diagnosed at 2 major tertiary hospitals in the Western region of Saudi Arabia and review all previously reported cases of RDD to shed light on the pattern of the disease in our society

## Methods

This retrospective study was carried out at King Abdulaziz University Hospital and King Faisal Specialist Hospital and Research Center, Jeddah, Saudi Arabia, 2 main referral hospitals in the western region of Saudi Arabia. Inclusion criteria included all cases diagnosed as RDD or sinus histiocytosis with massive lymphadenopathy between January 2001 to June 2021. The collected clinical data included age at presentation, gender, clinical features, treatment, and outcome. Periodic acid-Schiff (PAS) for fungi and Ziel-Neelsen stains (ZN) for acid-fast bacilli were carried out to rule out the infectious process. The immunohistochemistry was carried out using the avidin-biotin complex technique (ABC) with appropriate positive and negative controls. The basic immunohistochemistry panel carried out in all cases included CD45, CD68, S100, and CD1a. Further immunohistochemistry panel was added in selected cases. The study was approved by the Research Committee of the Biomedical Ethics Unit at our institution (Reference no.: 513-21). The study was carried out according to the principles of Helsinki Declaration.

### Statistical analysis

Chi-square test was used to assess the difference in the incidence of extranodal disease between male and female.

## Results

A total of 17 cases of RDD were retrieved. Four of those cases have been previously reported.^8-10^ The sites of the previously reported cases included a mass in the right eye, peribronchial LN, and 2 cases of the thoracic spine. The other 13 cases reported in this study are summarized in Table 1. There were 7 nodal RDD and 6 extranodal RDD. The male to female ratio was 1:1.2. All extranodal cases were isolated without LN involvement. The extranodal sites included the larynx, optic chiasm, brain, lumbar vertebrae, and left arm. All were adults with only one pediatric patient (4 years old). There was no statistically significant difference in the incidence of extranodal disease between males and females (*p*=0.76)

**Table 1:** - Summary of Rosai-Dorfman disease cases from 2 tertiary hospitals in the Western regions of Saudi Arabia.

**No.**	**Age/gender**	**Clinical presentation/site of biopsy**	**Treatment/procedure**	**Follow-up**	**Other involvement/associated findings**
1	50/female	Back pain/mass in lumbar vertebrae	Surgical resection	9 months/no recurrence	
2	24/female	Referred with optic chiasm tumor	Surgical biopsy	No available radiology	
3	44/female	Referred with right temporal brain tumor	Surgical resection	14 months/no recurrence	
4	56/female	left arm mass (soft tissue)	Surgical resection and steroid	60 months/no recurrence	Similar masses in the left shoulder and thigh
5	37/male	Hoarseness of voice/laryngeal mass	Surgical resection and steroid	Tumor recurrence occurred after 8 months	
6	39/male	Seizures/left parietal bone and dural lesion	Surgical resection	12 months/no recurrence	Subcortical white matter involvement
7	35/male	Submandibular mass lymph node enlargement	Surgical resection and steroid	24 months/no recurrence	
8	33/male	Cervical lymphadenopathy	Surgical resection	37 months/no recurrence	Dermatopathic lymphadenopathy
9	34/male	Cervical lymphadenopathy	Surgical resection and chemotherapy for lymphoma	Recurrence after 24 months in axillary and groin LN	Hodgkin’s lymphoma
10	10/female	Cervical lymphadenopathy	Surgical resection	9 months/no recurrence	
11	46/female	Cervical lymphadenopathy	Surgical resection	2 months/no recurrence	
12	40/female	Cervical lymphadenopathy	Surgical resection and chemotherapy for lymphoma	48 months/no recurrence	Hodgkin’s lymphoma
13	4/male	Cervical lymphadenopathy	Surgical resection	11 months/no recurrence	

No.: number, LN: lymph node

**Figure 1:**
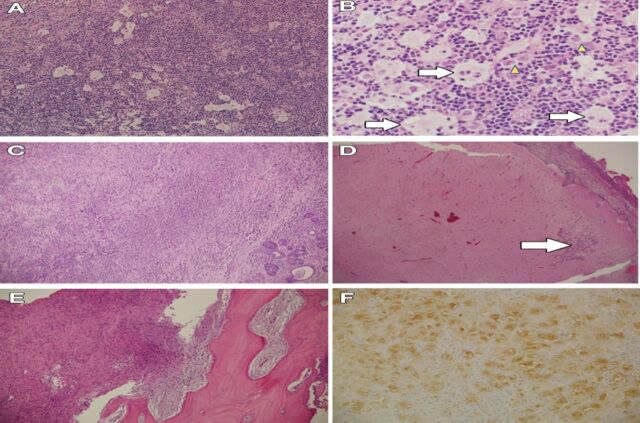
- Microscopic features of Rosai-Dorfman disease involving different organs. **A)** Patchy areas of Rosai-Dorfman disease (RDD) admixed with scattered Reed-Sternberg cells of classic Hodgkin’s lymphoma in the same lymph node (hematoxylin and eosin, 40×). **B)** Higher power; large histiocytes (arrows) with emperipolesis seen adjacent to scattered Reed-Sternberg cells of classic Hodgkin’s lymphoma (triangles) (hematoxylin and eosin, 400×). **C)** RDD involves the larynx and the infiltrate surrounding the seromucinous glands right lower quarter of the image (hematoxylin and eosin, 40×). **D)** RDD involves the dura (arrow) and extend to underlying glial tissue (arrow) (hematoxylin and eosin, 40×). **E)** RDD involves the bone (hematoxylin and eosin, 200×). **F)** S100 immunohistochemistry stain highlighted the histiocytes (400×).

All the cases showed the characteristic features of RDD with variable histiocytic proliferation revealing the pathognomonic emperipolesis, which was the presence of lymphocytes, plasma cells, and red blood cells within vacuoles in the cytoplasm of many histiocytes (Figure 1).

Two of the cases have been initially misdiagnosed in the outside institution; one was labeled as atypical infiltrate suspicious for lymphoma, and the other was called non-caseating granulomatous inflammation suggestive of sarcoidosis. A pathological review of these cases established the diagnosis of RDD.

Two cases were associated with Hodgkin’s lymphoma. In one patient, RDD was identified in subsequently enlarged LNs following the treatment of classic Hodgkin’s lymphoma. There were multiple enlarged LNs enlargement post-treatment with a clinical impression of lymphoma recurrence. In the initial true cut biopsy, this case was misdiagnosed as atypical cells suspicious for lymphoma recurrence due to the moderate atypia in the histiocytic proliferation and inconclusive immunohistochemistry. However, subsequent LN excision confirmed the diagnosis of RDD with no evidence of lymphoma. In the other patient, RDD was identified as focal involvement in the same LN that showed the classic Hodgkin’s lymphoma. Immunohistochemistry was very helpful in confirming the diagnosis, where the Reed-Sternberg cells were highlighted with immunoexpression of CD15, CD30, Fascin, and weakly for Pax-5, while the characteristic histiocytes of RDD were strongly positive for S100. In patient number 8, the enlarged lymph showed marked dermatopathic lymphadenopathy in addition to the characteristic pathological changes of RDD in the same LN. In patient number 7, the diagnosis was suggested based on the cytology specimen of fine needle aspiration and confirmed on the surgical excision.

Patient number 4 presented with left upper arm soft tissue mass that was associated with similar masses in the left shoulder and thigh. The patient was treated then by surgical excision and steroid therapy. There was one case of isolated laryngeal RDD. The patient presented with hoarseness of voice was treated with surgical excision and developed recurrence after 8 months. The patient was treated then by surgical excision and steroid therapy.

## Discussion

Rosai-Dorfman disease is a rare disease. Three cases involved the CNS in this series. The first case involved dura and extended to the underlying cortical tissue. The second case involved parietal bone, dural, and extended to subcortical white matter. The third case involved optic chiasm. Previously, we have reported 2 cases of RDD with involvement of spinal dura.^10^ Central nervous system involvement usually manifests as dura-based masses and should be considered in the differential diagnosis of a dura-based lesion such as meningiomas. Intracranial and spinal intramedullary involvement by RDD is extremely rare, and lesions can clinically mimic lymphomas. Intraventricular lesions have also been reported.^11^

There was one case of isolated laryngeal RDD. In a recent review by Wei et al,^12^ there were approximately 30 reported cases in English so far. Patients in those cases were treated in different ways. The largest case series was reported by Niu et al^13^ who reviewed 5 laryngeal RDD cases. Thalidomide treatment was found to be effective in the treatment of laryngeal RDD.^12^

One case in the current series was an osseous RDD involving lumbar vertebrae without involvement of spinal dura. The patient presented with lytic lesion. Primary osseous RDD is also very rare and is usually multifocal. The largest case series of osseous RDD was reported by Demicco et al.^14^ They reviewed 15 cases, and the lesions in their series arose in various anatomical locations. On radiological assessment, typically, these lesions tended to be lytic with well-defined sclerotic margins.^14^ Bone RDD must be differentiated from the pathological mimics of bone lesions, especially in Erdheim-Chester disease.

Only one soft tissue RDD was identified in this study, in the left upper arm. In this patient, the disease was multifocal, with similar masses identified in the left shoulder, and thigh. According to the literature, skin and soft tissue represented the most reported sites of extranodal involvement by RDD. However, a cutaneous disease without lymphadenopathy is extremely rare.^2,15^ Skin lesions must be differentiated from different cutaneous mimics, including histiocytosis, lymphoma, sarcoidosis, and infectious processes.^15^

A review of the English literature revealed 12 RDD cases reported from Saudi Arabia and is summarized in Table 2.8-10,16-22 In those cases, the age range was between 10 months and 53 years. There were 8 males and 4 females. The tumor site included LNs, cheek, cornea, orbit, and thoracic spine. All patients were adults except one case reported in infancy.^22^ Three of the cases were reported in association with other diseases, including sickle cell disease and eczema, polyarthritis, and Sjogren’s syndrome.^16,18,23^ In those cases, patients were treated by surgical excision alone, surgical excision with systemic steroid, surgery with local steroid, surgery with radiation, steroid alone, and steroid with azathioprine.^8-10,16-18,20-24^

**Table 2:** - Summary of the Rosai-Dorfman disease cases reported from Saudi Arabia.

**Studies**	**Age/gender**	**Site of biopsy**	**Other involvement**	**Treatment**	**Follow-up**	**Associated findings**
Maklad et al^21^	26/male	Right cheek swelling	left upper eyelid swelling	Surgery, steroids, chemotherapy, and radiation	one year/recurrence	
JI Wani^24^	25/female	Lymph node	Generalized lymphadenopathy with hepatosplenomegaly	Steroids and azathioprine	Died after 4 months	
Alharbi et al^16^	29/male	Corneal mass	Isolated, no other involvement	Surgical resection and local steroids	one year/no recurrence	sickle cell disease and eczema
Al-Jahdali et al^17^	26/male	Hilar lymph node	Isolated, no other involvement	Surgical resection and steroids	6 years/no recurrence	
Baeesa et al^10^	50/female	thoracic spine lesions	Isolated, no other involvement	Surgical resection and steroid	4.5 years/no recurrence	
	35/male	thoracic spine lesions		Surgical resection, followed by radiation for recurrence after 6 months	5 years/no recurrence	
Al-Maghrabi et al^9^	52/female	Peribronchial mass	Isolated, no other involvement, obliterating the right main and upper lobe bronchus	Surgical resection	6 months/no recurrence	
Al-Maghrabi et al^8^	53/male	Mass in right eye	Isolated, no other involvement	Surgical resection	2 years/no recurrence	
Medhat et al^23^	25/female	Para-aortic lymph node	Hepatosplenomegaly, enlarged para-aortic, portahepatic, paratracheal, subcarinal, and cervical lymph nodes	Steroids	Not available	polyarthritis
AlKuwaity et al^18^	40/male	Submandibular lymph node	Isolated, no other involvement	Steroid and azathioprine	2 months/no recurrence	Parotid (Sjogren’s syndrome)
Fathala et al^20^	37/male	Cervical lymph node	Bilateral cervical and inguinal lymphadenopathy	Steroid	5 months/no recurrence	
Al-Qahtani et al^22^	10 months/male	Cervical lymph node	Isolated, no other involvement	Surgical resection and steroids	2 years/no recurrence	

Nodal RDD frequently mimics other diseases clinically. Because RDD is a rare disease and has various clinical presentations, it can mimic other causes of lymphadenopathy. All 7 cases of nodal RDD in this series were samples for the clinical suspicion of lymphoid neoplasm. Pathological examination is important to distinguish RDD from lymphoma because they are completely different entities. The pathological mimics of RDD include malignancy like lymphoma, histiocytic sarcoma, metastasis or other benign like tuberculosis, sarcoidosis, immunoglobulin G4 (IgG4) related disease, and Erdheim-Chester disease. Two of the cases in the current study have been initially pathologically misdiagnosed outside the institutions, one was labeled as atypical infiltrate suspicious for lymphoma, and the other was called non-caseating granulomatous inflammation suggestive of sarcoidosis. The misdiagnosis of RDD as another entity was well known in the literature. Rosai-Dorfman disease has been misdiagnosed histologically or cytologically as meningioma, rhinoscleroma, toxoplasma lymphadenitis, sarcoidosis, tuberculosis, and lymphoma.^25^ Careful morphological assessment and proper immunohistochemistry interpretation usually prevent misdiagnosis. Rosai-Dorfman disease has been reported in association with other histiocytic disorders, such as Langerhans cell histiocytosis.^26^ It can be associated with both Hodgkin’s and non-Hodgkin’s lymphoma, including mantle cell lymphoma, peripheral T-cell lymphoma, small lymphocytic lymphoma, extranodal marginal zone lymphoma, and large-cell anaplastic lymphoma.^27,28^ Rosai-Dorfman disease can precede, coincide with, or follow lymphoma diagnosis.^27^ Rosai-Dorfman disease and lymphoma may involve the same or different anatomic locations. Therefore, the diagnosis of RDD in LN should raise possible associated lymphoma in the same LN or in different anatomical sites.

Among the 25 cases reported from Saudi Arabia (including the patients in this series), 14 were nodal, and 11 cases extranodal with CNS and spine represented the most common site (total of 5 cases). The prognosis in those patients reported from Saudi Arabia was generally very good, with only 3 recurrences of disease and one fatal case, where the patient failed treatment, deteriorated clinically, and died within 4 months after diagnosis.^24^ Only 2 cutaneous RDD were identified, which suggest that the cutaneous form of RDD is rare among Saudis compared to Asians.

The pathogenesis of RDD is still unclear and likely multifactorial. The relation of RDD with different viruses is a controversial issue. Different viruses have been reported associated with RDD, including Epstein-Barr virus, human herpesvirus-6, and parvovirus B19 infection.^29-31^ The possible association between RDD and different autoimmune diseases, IgG4-related diseases, have been also reported.^32,33^ Recent studies have shown Kirsten rat sarcoma viral oncogene homolog (KRAS) and mitogen-activated protein kinase 1 (MAP2K1) mutations in some cases of RDD, suggesting that at least a subset of RDD cases may be clonal lesions, which may open new channels for targeted therapy, particularly in refractory cases.^34-37^

Pathologically, RDD is characterized by histiocytic proliferation that may show mild to moderate cytologic atypia with nuclear pleomorphism and prominent nucleoli. The pathognomonic histopathological feature is emperipolesis, which is the presence of lymphocytes, plasma cells, red blood cells, and less frequently neutrophils within vacuoles in the cytoplasm of many histiocytes. Although emperipolesis is considered a characteristic feature of RDD, it can rarely be seen in association with lymphomas, with few cases reported in the literature.^38^ Awareness of this fact is important for the pathologist to avoid misdiagnosis of lymphomas as RDD with subsequent delayed management. Rosai-Dorfman disease may have some overlapping features with IgG4-related disease. However, it lacks other histologic criteria for IgG4-related disease, mainly storiform fibrosis and obliterative phlebitis.

When RDD involves the LN, the germinal centers are usually atrophic, compressed, or lacking in most cases, and the paracortex is attenuated due to the compression effect of the expanded sinusoids. The histiocytes in RDD classically are positive for S100 and CD68, but they lack markers of Langerhans cells (CD1a and langerin) or dendritic cells (CD21, CD23, and CD35). This immunohistochemistry profile is very helpful in differentiating this entity from other histiocytic diseases.

Surgery, steroid, chemotherapy, methotrexate, azathioprine, thalidomide, and radiation have been used to treat RDD. While the optimal treatment remains controversial, surgical resection is generally curative. Abla et al^37^ and Salama et al^35^ reported guidelines for clinical evaluation with treatment recommendations for a patient with RDD based on their clinical experience and review of the literature. Targeted therapy with mitogen-activated protein kinase inhibitors is under investigation in those with MAPK/ERK pathway alterations.^36,39^ In most cases, RDD has a benign course and treatment is not required. Treatment is indicated for patients with extranodal RDD affecting vital organs or those with nodal RDD associated with serious complications.

Rosai-Dorfman disease reportedly has a benign prognosis with good response to treatment. However, it is unpredictable. Some patients had progressive disease and died.^40^ In others, the disease seemed to regress. Fatalities were reported to be in the range of 5-10%.^36^ Because of the rarity of this disease, reports of even small patient series will highlight more on this entity in particularly the extranodal type and provided more information on the clinical presentation and management options.

### Study limitations

The study covered the disease pattern in only 2 hospitals and analyzed only the reported cases from different institutions in Saudi Arabia. The exact pattern of the disease can be better evaluated on the national wide study. Multicenter prospective studies are probably the best to evaluate treatment options and improve outcomes.

In conclusion, this is a series of RDD reported from Saudi Arabia. Awareness of this entity is important for pathologists to avoid misdiagnosis. The prognosis is generally good with rare recurrence or persistent disease.
